# Understanding Variable Motor Responses to Direct Electrical Stimulation of the Human Motor Cortex During Brain Surgery

**DOI:** 10.3389/fsurg.2021.730367

**Published:** 2021-10-01

**Authors:** Daniel M. Aaronson, Eduardo Martinez Del Campo, Timothy F. Boerger, Brian Conway, Sarah Cornell, Matthew Tate, Wade M. Mueller, Edward F. Chang, Max O. Krucoff

**Affiliations:** ^1^Department of Neurosurgery, Medical College of Wisconsin, Milwaukee, WI, United States; ^2^Medical College of Wisconsin, Milwaukee, WI, United States; ^3^Department of Neurosurgery, Feinberg School of Medicine, Northwestern University, Chicago, IL, United States; ^4^Department of Neurosurgery, University of California, San Francisco, San Francisco, CA, United States; ^5^Department of Biomedical Engineering, Marquette University, Milwaukee, WI, United States

**Keywords:** brain mapping, motor cortex, cortical plasticity, brain stimulation, direct electrical stimulation (DES)

## Abstract

Direct electrical stimulation of the brain is the gold standard technique used to define functional-anatomical relationships during neurosurgical procedures. Areas that respond to stimulation are considered “critical nodes” of circuits that must remain intact for the subject to maintain the ability to perform certain functions, like moving and speaking. Despite its routine use, the neurophysiology underlying downstream motor responses to electrical stimulation of the brain, such as muscle contraction or movement arrest, is poorly understood. Furthermore, varying and sometimes counterintuitive responses can be seen depending on how and where the stimulation is applied, even within the human primary motor cortex. Therefore, here we review relevant neuroanatomy of the human motor system, provide a brief historical perspective on electrical brain stimulation, explore mechanistic variations in stimulation applications, examine neurophysiological properties of different parts of the motor system, and suggest areas of future research that can promote a better understanding of the interaction between electrical stimulation of the brain and its function.

## Introduction

The use of direct electrical stimulation (DES) of the human brain to define functional-anatomical relationships dates back to the very beginnings of modern neurosurgery (1). Currently, it is the gold standard technique used to map the brain's somatotopy and reduce the rate of postoperative neurological deficits in glioma and epilepsy surgeries (2). Areas of the brain that produce a response upon stimulation are considered gateway “critical nodes” into cerebral circuits that control functional movement and language. Although DES is used in neurosurgical procedures across the world (3–5), when a response is generated, the pathway from stimulus to effect is generally poorly understood. The neurophysiology of underlying how an electrical stimulus interacts with a given population of neurons can vary widely. Specifically, within the motor cortex, when stimulation induces local action potentials, the circuit modulation and downstream effects can result in silence, muscle activation, or motor inhibition. Over the course of the last century and a half, much has been learned about the mechanisms and neurophysiological properties of the motor system, cortical circuitry, and motor control. Despite these advances, there is still a limited understanding of how stimulation responses vary across individuals, pathologies, and stimulation parameters. Therefore, here we review relevant neuroanatomy of the human motor system, provide a brief historical perspective on electrical brain stimulation, explore mechanistic variations in stimulation applications, examine neurophysiological properties of different parts of the motor system, and suggest areas of future research that can promote a better understanding of the interaction between electrical stimulation of the brain and its function.

## Manuscript

### A Brief History of Direct Electrical Brain Stimulation

Direct electrical stimulation of cortical structures to investigate anatomical function has been used since the second half of the 19^th^ century in animals (6). Although the technology at the time was somewhat crude [i.e., the intensity of the stimulation was measured by subjective sensation when applied to the experimenter's tongue (7)], it was not long before translation to the first trial of cortical stimulation of a human by Robert Bartholow in 1874 (8). In this famous case of a patient with basal cell carcinoma and exposed brain, Bartholow inserted electrodes into parenchyma [likely Brodmann area 7 bilaterally (9)] and elicited contralateral muscle contractions reliably from both sides of the brain. Around the turn of the 20th century, this knowledge was put to practical neurosurgical applications by Horsley, Bidwell, Krause, and Cushing (1, 10–12), who continued to use this technique to expand the understanding of brain function over many years.

**Figure 1:**
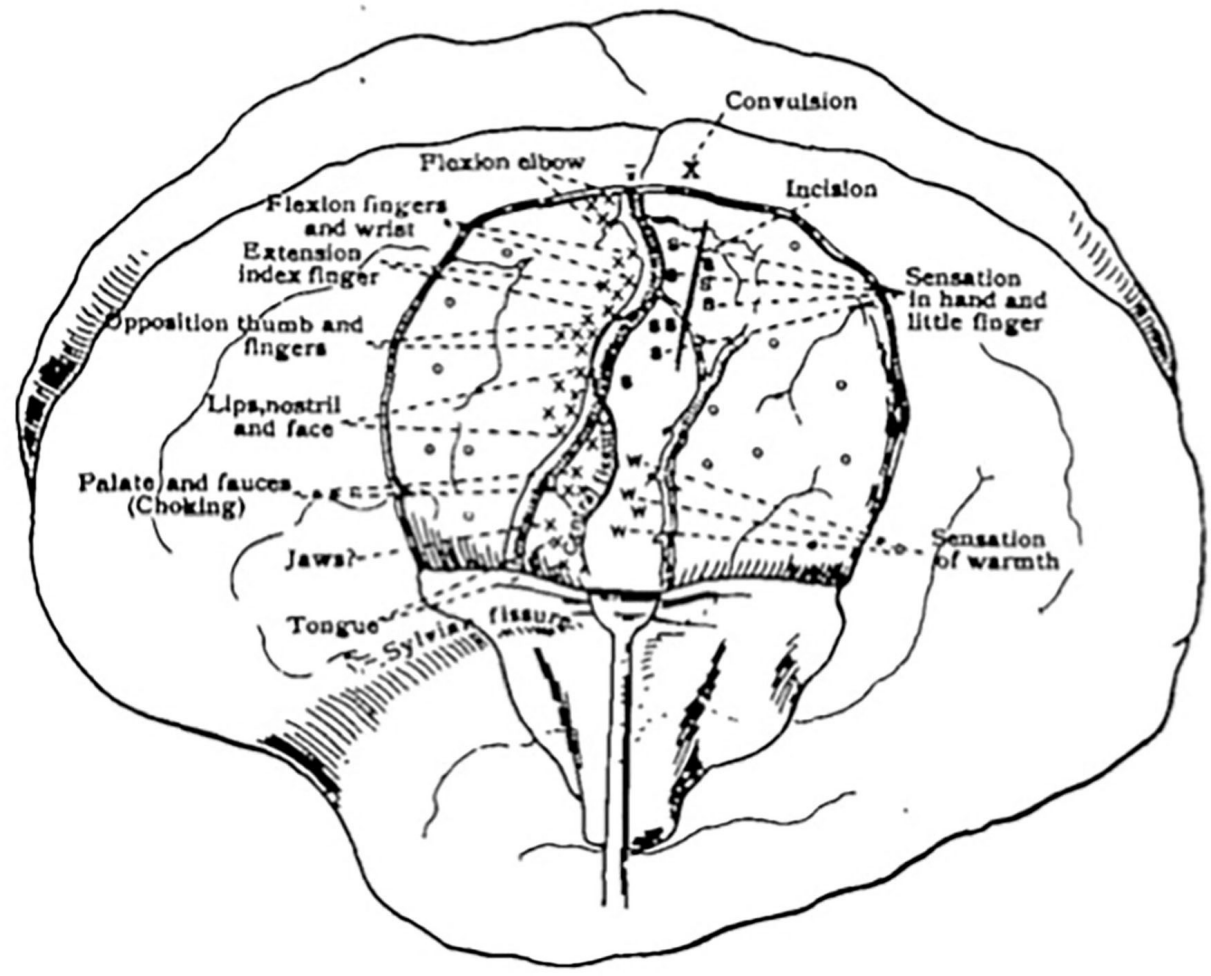
First sketch mapping motor and sensory responses during an awake craniotomy. Reprinted from Cushing H., A note upon the faradic stimulation of the postcentral gyrus in conscious patients, Brain, 1909;32(1):44–53 by permission of Oxford University Press.

Initially, there was debate among scholars as to which areas of the cortex contributed to motor and sensory function and which areas did not. Some firmly believed motor and sensory function to be combined as one sensorimotor region, while others believed motor localization to be distinct and belong purely to the region anterior to the central sulcus (13, 14). A significant shift in theory was noted after the work of Grünbaum and Sherrington (13), which led to more a concrete model of an anatomically distinct pre-Rolandic motor cortex and post-Rolandic sensory cortex (15). Their work, along with contributions from Krause, was among the first to illustrate a somatotopic map of the motor cortex (12). Cushing reported using DES in his anesthetized patients (1) as early as 1902 (16), and soon after progressed to using the technique on awake patients, allowing him to interpret sensory information in the post-central gyrus as well (Figure 1).

In the mid-twentieth century, Penfield described the density of cortical organization through the visual representation of the sensorimotor homunculus (13). His work alongside Rasmussen continued to examine localization of cortical functions (17), and his work with Welch expanded knowledge of planning of motor function in the anterior supplementary motor area (SMA), defining a region involved in complex movements and initiation of movements (18).

When Penfield and Rasmussen reported negative effects (e.g., inhibition and muscle relaxation) upon stimulating certain areas of the motor cortex, the nature of Brodmann area 6 began to be questioned once again (17). This finding led some to think motor planning and inhibition may also be involved in this region. Along the way, the model of the motor cortex began to evolve from a simplistic, positive response area whereby “a chain of neurons is activated and an effective impulse passes out to the periphery” (13), to include areas of planning and areas of negative responses resulting in motor inhibition (which will be further described later in this paper).

In sum, DES of the cortex has been the primary tool used to define cerebral anatomical-functional relationships (19) from Penfield's work on sensorimotor systems (20) to Ojemann's studies on language (21). In addition to its use as an investigational tool, it has also been a critical surgical tool used to outline functional-anatomical somatotopy and predict and minimize post-operative deficits. Throughout the 20th century and beyond, DES has become standard of care for patients undergoing resections of brain tumors (22) and epilepsy lesions (23) in eloquent motor and/or language systems (24–38).

### Relevant Anatomy of the Human Primary Motor System

**Figure 2:**
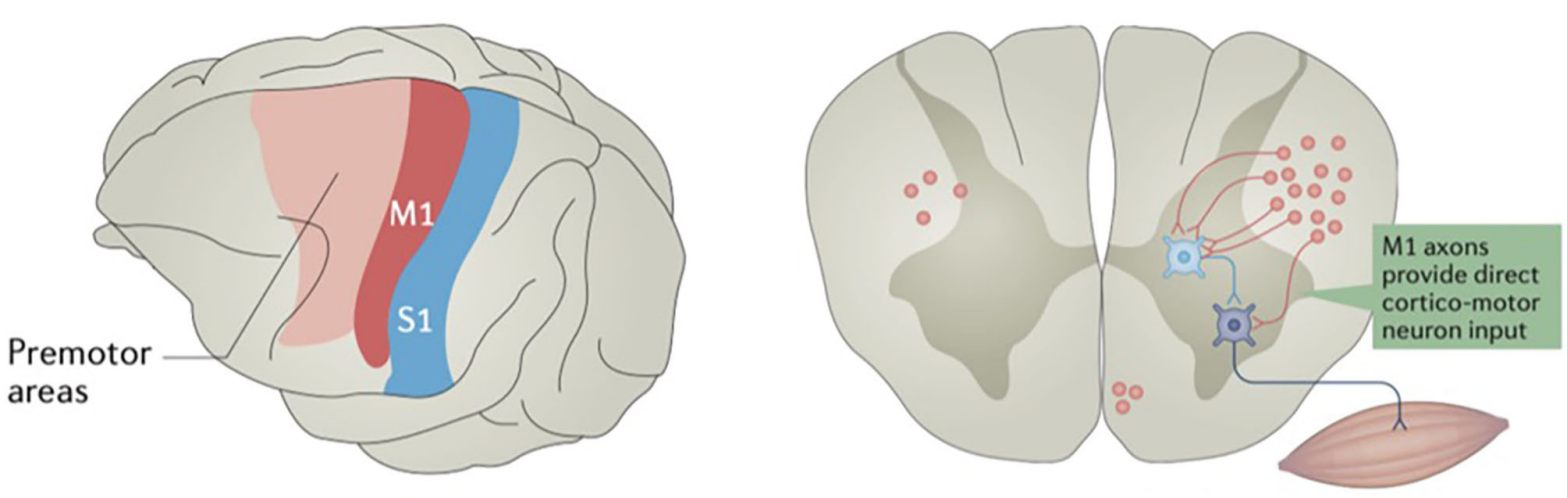
Some motor cortex neurons terminate on spinal cord interneurons, while others terminate directly on motor neurons. The primary motor cortex (M1) is in red, and primary somatosensory cortex (S1) is in blue. Reprinted by permission from Springer Nature Customer Service Centre GmbH: Springer Nature, Nature Reviews: Neuroscience, Motor cortex—to act or not to act? Christian Laut Ebbesen, et al., 2017.

Neuronal cell bodies located in layer 5 of the primary motor cortex have axons that project down the corticobulbar and corticospinal tracts to either synapse directly onto motor neurons or interneurons of the brainstem and spinal cord (Figure 2) (39). Layer 5 neurons also have connections with other cortical and subcortical structures, ranging from association fibers to the somatosensory cortex to outputs to the direct and indirect pathways of the basal ganglia (40–42). Because of this complex network of connections, the sum of direct output from the primary motor cortex is not exclusively excitatory. Other nearby anterior motor regions, such as the SMA and premotor areas, as well as some posterior parietal regions, have been shown to be linked to generating intent to perform an action and ordering complex motor movements later effectuated by the motor cortex (18, 43–46). Additionally, there is evidence that the more anterior “planning regions” may have their own direct influence on the spinal cord (47), perhaps in parallel to the corticospinal tract (48). This evidence is consistent with primate experiments supporting a model where primary motor cortex neurons more directly encode muscle activity, or kinetics, to a greater extent than limb position or velocity, or kinematics (49). While the concept of these “anterior planning regions” is generally accepted, this is not a strict, linear hierarchy, as other frontal and parietal areas have been shown to participate in subcortical motor networks (48).

The primary motor cortex has been described as a discrete functional-anatomical interface along a unimodal gradient (50), meaning it is a cortical region with a somatotopic organization where movement intention is translated into action. This unimodal network gradient description may account for the more homogeneous, reproducible results of direct electrical stimulation on the motor system (51) when compared with more transmodal networks like emotion and cognition, for example (50). However, studies of motor connectivity have shown both interindividual variability and plasticity in recovery from deficits (52), suggesting a neural network model that can modulate function in a dynamic fashion (19). This has been demonstrated specifically in the primary motor cortex in patients with infiltrating tumors (53, 54). In other words, in a static model, input A may always lead to output B; however, in a dynamic model, input A may lead to output B, or may lead to output C, depending on inputs from other systems. Stimulation of the motor cortex to induce plasticity has also begun to be explored, showing that durable plastic changes in the motor cortex may also be artificially constructed for therapeutic applications (55).

### Modern Neurostimulation Techniques and Related Neurophysiology

DES predominantly affects axons (56) by inducing a modulation in membrane potential through a directly applied electric current (57) that triggers a response. The downstream result of this stimulation can vary significantly and depends on a number of factors. Modifiable factors include the stimulating parameters (i.e., pulse type, width, frequency, and intensity) (58), probe configuration (i.e., monopolar or bipolar) (4), and anatomical location. Alterations in these variables can affect which cells are stimulated, to what extent they are stimulated, and what their response to that stimulation might be.

#### Stimulation Parameters

The effect of varying stimulation parameters on downstream motor function has not been systematically tested in humans to our knowledge. However, many studies do provide some insight into how certain stimulation parameters effect downstream effects. For example, stimulation amplitude can alter how cells behave by inducing hyperpolarization from large current delivery (59), and, as the current spreads from an electrode and charge drops over distance, the net effect may be hyperpolarization in the immediate vicinity of the electrode and initiation of action potentials at greater distances. Independent of current amplitude, inhibitory effects may also be induced through indirect signal propagation through cortical interneurons (60).

In addition to current amplitude, changing the frequency of stimulation has been shown to modulate neuronal activity. One study (58) found that lower frequency stimulation (i.e., 10–50 Hz) was more likely to cause neuronal suppression, whereas higher frequency stimulation (i.e., 100–200 Hz) was more likely to lead to neuronal activation. The authors of this study proposed that lower frequency stimulation may activate passing axons, whereas higher frequency stimulation activates cell bodies, thus accounting for the difference. It should be noted, however, that the neuronal activation measured in this study was high frequency activation [HFA] of neurons, not downstream motor function (i.e., hand movement or speech activation).

Environmental factors may also alter the interface through which the current is delivered. For example, the pia matter itself has significant resistance and capacitance that can alter stimulation, which changes over the amount of time exposed to air (61). In theory, these changes could lead to the delivery of different currents over the duration of an operation despite using the same stimulation parameters at the same location. While the intention may be to stimulate a focal region only, the end result may be the stimulation of “an unknown number and unknown kinds of cells at unknown locations in the vicinity of the electrode” (59).

#### Bipolar vs. Monopolar Stimulation

The original technique introduced by Penfield involving bipolar stimulation at a frequency between 50–60 Hz delivered in long trains (1–4 s) of biphasic pulses remains the gold standard in neurosurgical practice (24, 35, 62). More recent developments include a monopolar technique first described by Taniguchi et al. in 1993 that instead uses a train of 5–10 short pulses (10–18 ms) at higher frequencies of 250–500 Hz (63, 64). Also known as the “train-of-five,” this technique has been popularized in recent years by Szelényi et al. (64) and Bello et al. (65), as it has shown higher sensitivity in identifying motor pathways (64, 66–68) with equal safety and efficacy when compared to bipolar stimulation techniques (69). Some surgeons have chosen to combine both bipolar stimulation, for maximal definitive resolution, with monopolar stimulation, for sensitivity and estimation of distance to motor pathways, to maximize the advantages from both modalities (64, 66), and the addition of concurrent motor evoked potential monitoring has been termed “triple motor mapping” (66).

While traditional DES implies cortical surface stimulation, the advent of subcortical mapping in the late 20^th^ century has also proven quite useful (26, 27, 30, 33, 48, 62, 70, 71). Subcortical mapping evaluates for white matter involvement throughout the duration of the surgery, and allows the surgeon to safely resect tissue deep to the cortex without violating irreparable tracts. A recent method introduced by Yamaguchi et al. (72) utilizes a neuronavigated bipolar stimulator with needle-tipped electrodes that can be inserted directly into subcortical tissue. This stimulator aims to minimize conduction through heterogenous tissue which may alter delivery of the stimulation current. The stimulator was coupled with plastic tubes which could be left *in situ* as “fence post markers” to aid in establishing neuronavigated and stimulation-confirmed white matter borders prior to brain shift from tumor resection.

### Variable Downstream Motor Effects

Motor effects can be monitored by visual observation of motor end phenomenon or continuous electromyography (EMG) monitoring (73). Patients must not be chemically paralyzed to observe end motor phenomenon, and anesthetic agents and doses can play a role in the sensitivity and success of motor mapping (74–77).

Historically, responses to direct stimulation of the cortex have been divided into two broad categories, as described by Duffau (19):

1. A “positive motor response” (PMR), in which a neurologic downstream effect is actuated in a resting state, such as a sum excitatory signal causing muscle contraction.
2. A “negative motor response” (NMR), in which there is inhibition of an intended action, such as induced aphasia or arrest of a repeated action.

When DES results in an NMR, or inhibition of movement without loss of consciousness, this stimulated cortical region is referred to as a negative motor area (NMA) (78). This phenomenon is distinct from activation and contraction of an opposing muscle group, which would still be considered a PMR. While NMAs were previously thought to be either distributed widely along the lateral aspect of a given cortical hemisphere (79–81) or somatotopically located in the inferior frontal gyrus (82), more recent work shows NMAs to be more reliably located in several areas within the precentral gyrus (83), although not exclusively (84). In general, NMAs appear to localize in two main regions, a more medial region which includes the SMA and pre-SMA regions, and a more lateral region which includes the inferior frontal gyrus and the premotor cortex (80). Additional NMAs with clinical relevance include those in the parietal lobe, stimulation of which can lead to hand apraxia (85), for which specific hand-motor tasks can be monitored during DES to avoid post-operative deficits (86).

The mechanism by which NMRs are generated is not yet understood, and there are different viewpoints represented in the literature. Mikuni et al. (79) suggests these NMRs represent external disruption of physiologically excitatory pathways. A similar mechanism has been proposed by Duffau et al., who categorized these NMRs as a “second intermediate level” of functional disturbance due to DES, namely that the task inhibition is due to disruption of a subcircuit network (19). Others have postulated the NMRs represent activation of naturally encoded inhibitory pathways within, or relating to, the motor cortex (87). This has been supported by fMRI studies which have shown motor region activation patterns for muscle relaxation to be similar to activation patterns for muscle contraction (88), with accompanying evidence that these processes are driven by an excitatory, active process as opposed to neuronal suppression (89, 90).

In addition to underlying anatomical physiology, widespread heterogeneity in how DES has been applied may account for some differences in results. In Mikuni et al.'s study, for example, stimulation was performed at 50 Hz in square waves of alternating polarity with 0.3 ms duration for 1 to 5 s between subdural electrodes with intensity ranging between 2 and 15 mA. They found regions where a low stimulation intensity would trigger a NMRs, while higher intensity stimulation in the same region could then induce a PMR (79). More recent studies by Rech et al., however, only stimulated at lower intensities (on the range of 2 mA) due to time constraints at 60 Hz with biphasic current and 1 ms pulse width for 4 s via a bipolar electrode with tip width set at 5 mm, finding no NMAs that eventually produced a PMR (83). This may be due to modulation of the neuronal population recruited in the NMA based on electrophysiological response or could alternatively be explained by a wider recruitment field including PMR-controlling neurons with higher intensity, as increased current travels over larger distances. Ultimately, the gap in understanding the effects of varying stimulation parameters in certain anatomical locations on downstream motor systems outlines the need for future studies in this area.

### Translation to Clinical Practice

While DES is undeniably the goal standard to intraoperatively map functional-anatomical somatotopy of the motor system, current DES techniques vary widely. Controversies include awake vs. asleep mapping, complex tasks (i.e., apraxia) vs. simple motor response mapping, bipolar vs. monopolar stimulation, high vs. low frequency stimulation, continuous motor evoked potentials vs. repeated intraoperative stimulation, length and type of stimulus pattern, and gray vs. subcortical mapping. As discussed above, there is a general movement to combine these modalities into more nuanced mapping/resection strategies, as opposed to using one vs. the other. While bipolar stimulation at 50–60Hz has been the most widely employed method for mapping the motor cortex, advantages to high frequency monopolar stimulation may include fewer intraoperative seizures and increased sensitivity (64, 66–68). While there is evidence to support this claim, it has not yet been widely adopted as a cortical stimulation technique. When performing subcortical stimulation in descending motor pathways, the use of a train of multiple high-frequency monopolar stimulation pulses at 250–500 Hz may afford the surgeon similar advantages. In one series, the addition of monopolar stimulation to standard bipolar stimulation for the subcortical regions increased identification of descending motor pathways from 30 to 86.4% (66), similar to Szelényi et al.'s work which improved sensitivity from 54% using bipolar to 92% using monopolar stimulation (65). As mentioned prior, some have chosen to combine bipolar and monopolar stimulation with concurrent motor evoked potential monitoring, termed “triple motor mapping” (66).

Advantages to awake intraoperative mapping include surgeon confidence in the patient's neurological status, fewer intraoperative technical nuances obscuring the meaning of signal loss, and the ability to map more complex motor, cognitive, sensory, and speech-language systems. Disadvantages include patient discomfort and false negative exam responses due to, for example, development of an intraoperative SMA syndrome leading to a smaller extent of resection. Multiple studies have sought to evaluate outcome differences in awake vs. asleep motor mapping; however, the amalgamation of the available evidence does not support one technique over the other. Ultimately, decisions on how to intraoperatively map the motor system across neurosurgical operating rooms will depend on the specifics of case, including surgeon experience, patient goals and abilities, and other necessary functional assessments (i.e., language). The authors do generally advocate a trend toward using bipolar stimulation on the cortex, high frequency monopolar stimulation on the subcortical white matter, and continuous motor evoked potentials during resection wherever possible. Also, for pure motor cases, the authors generally prefer asleep mapping to prevent false positive exam changes from phenomenae such as SMA syndrome that might prematurely conclude the surgery, with the caveat of trending toward awake mapping when more complex task monitoring is needed (i.e., apraxia and/or speech-language).

## Conclusion

Systematic stimulation parameter testing in the motor cortex is needed. Additionally, there is much discrepancy in both the definition and locations of NMAs, and developing more objective ways of measuring and detailing motor function and inhibitory effects during stimulation would make this type of testing more broadly applicable. Also, the application of more chronic types of stimulation in ambulatory patients and their potential to modulate neuronal circuits are becoming more widely available (91). With the development of FDA-approved, chronically implanted devices that can both sense neuronal signals and stimulate the cortex, new ambulatory recordings and stimulation-plasticity induction techniques may follow (92, 93). Additionally, progress is being made in non-invasive techniques of cortical stimulation, specifically with navigated transcranial magnetic stimulation (TMS), which is being used in certain centers to augment and/or predict DES findings (94).

DES is an important tool to investigate anatomical-functional relationships in neurosurgical practice. Electrical stimulation of the motor cortex in the literature is heterogeneously applied, and care must be taken in interpreting results as differences in stimulation techniques, anatomical applications, underlying pathologies, and patient populations may impact the results. As described above, stimulation parameters, recruitment of nearby cells, membrane potential changes, and the parts of the cell stimulated can all change the functional outcomes of a given stimulated region. Furthermore, motor circuits are not a simple unimodal hierarchy of neurons. DES may effectuate inhibitory subcortical interneurons, modulation circuits, or spinal interneuron circuits as well as corticospinal tracts. Lastly, stimulation can induce both positive and negative motor responses, depending on both the stimulation location and input parameters. Variance in a multitude of these parameters may lead to alterations in downstream motor outcome, which may also change over time due transitory changes in connectivity across multiple neural networks. Therefore, the motor cortex may be best described as “an input gate into a large-scale network” (95), rather than as an isolated discrete functional site. Future studies systematically varying stimulation parameters, anatomical locations, and downstream effects are needed.

## Author Contributions

DA and MK contributed to the conception, design and wrote sections of the manuscript. DA wrote the first draft. All authors contributed to manuscript revision, read, and approved the submitted version.

## Conflict of Interest

The authors declare that the research was conducted in the absence of any commercial or financial relationships that could be construed as a potential conflict of interest.

## Publisher's Note

All claims expressed in this article are solely those of the authors and do not necessarily represent those of their affiliated organizations, or those of the publisher, the editors and the reviewers. Any product that may be evaluated in this article, or claim that may be made by its manufacturer, is not guaranteed or endorsed by the publisher.

## Abbreviations

DES: direct electrical stimulation
SMA: supplementary motor area
EMG: electromyography
NMR: negative motor response
NMA: negative motor area
PMR: positive motor response.
